# Invertebrate community response to fire and rodent activity in the Mojave and Great Basin Deserts

**DOI:** 10.1002/ece3.5189

**Published:** 2019-04-26

**Authors:** Joshua D. Day, Jackson H. Birrell, Tyson J. Terry, Amy Clark, Phil Allen, Samuel B. St. Clair

**Affiliations:** ^1^ Department of Plant and Wildlife Sciences Brigham Young University Provo Utah

**Keywords:** ants, *Bromus tectorum*, insects, invasion, small mammals

## Abstract

Recent increases in the frequency and size of desert wildfires bring into question the impacts of fire on desert invertebrate communities. Furthermore, consumer communities can strongly impact invertebrates through predation and top‐down effects on plant community assembly. We experimentally applied burn and rodent exclusion treatments in a full factorial design at sites in both the Mojave and Great Basin deserts to examine the impact that fire and rodent consumers have on invertebrate communities. Pitfall traps were used to survey invertebrates from April through September 2016 to determine changes in abundance, richness, and diversity of invertebrate communities in response to fire and rodent treatments. Generally speaking, rodent exclusion had very little effect on invertebrate abundance or ant abundance, richness or diversity. The one exception was ant abundance, which was higher in rodent access plots than in rodent exclusion plots in June 2016, but only at the Great Basin site. Fire had little effect on the abundances of invertebrate groups at either desert site, with the exception of a negative effect on flying‐forager abundance at our Great Basin site. However, fire reduced ant species richness and Shannon's diversity at both desert sites. Fire did appear to indirectly affect ant community composition by altering plant community composition. Structural equation models suggest that fire increased invasive plant cover, which negatively impacted ant species richness and Shannon's diversity, a pattern that was consistent at both desert sites. These results suggest that invertebrate communities demonstrate some resilience to fire and invasions but increasing fire and spread of invasive due to invasive grass fire cycles may put increasing pressure on the stability of invertebrate communities.

## 1 INTRODUCTION

Disturbance and exotic plant invasion are an increasing threat to global biodiversity (Brooks et al., 2004; D'Antonio & Vitousek, 1992).Changes in disturbance regimes due to human activities can have negative impacts on ecosystem biodiversity (Hobbs & Huenneke, 1992). Historically, fire has played a minor role in plant community succession in North American deserts. However, exotic annual grasses have altered fire regimes by increasing the size, frequency, and severity of fires (Brooks et al., 2004; Brooks & Matchett, 2006), which could have long‐term effects on the stability and biodiversity of these systems.

Invertebrates make up a large proportion of ecosystem diversity (May, 1988) and provide a wide range of ecosystem functions. Invertebrates often have specialized relationships with plants, vertebrates, and microbes. Invertebrates are critical to food webs in serving as prey for many vertebrate species and have important interactions with plants through herbivory, seed dispersal, and pollination. Many invertebrates have small home ranges, making them less able to escape unfavorable changes in their environment. These qualities make invertebrates good indicators of ecosystem function and resilience (Andersen, 1990; Lavelle et al., 2006; Majer, 1983). Ants (Hymenoptera: Formicidae) are particularly good indicators of ecosystem stability (Andersen, 1997) because they are among the most abundant and diverse group of invertebrates, and occupy a variety of specialized niches across multiple trophic levels (Majer, 1983).

The recent increase in the frequency and size of desert wildfires (Brooks et al., 2004) brings into question the direct and indirect impacts of fire on desert insect communities. Direct fire mortality is influenced by the degree of exposure and the mobility of the species or life stage (Swengel, 2001). Rice (1932) and Morris (1971) show that mortality can often continue to occur postfire from starvation and exposure while others report shifts in insect abundance and diversity after repeat burns (Wright & Samways, 1998, 1999). Flying insects and other highly mobile insects are often the first to recolonize into burned landscapes (Swengel, 2001). Grasshoppers have been shown to increase in abundance in burned areas (Lamotte, 1975); however, grasshopper richness is usually lower in frequently burned areas (Evans, 1984, 1988). Evans (1984, 1988) found that forb‐feeding grasshopper richness declined in more frequently burned areas because of fewer forbs in those areas, and grass‐feeding grasshoppers increased because of relatively higher grass cover in burned areas. Insect species that require a specific plant community structure that does not reoccur in the first few years after fire can lose resource availability for generations, and, if fires are too frequent, this can dramatically reduce their population size (Wright & Samways, 1998, 1999). Fire tends to favor some ant species (Holbrook et al., 2016), while reducing overall ant species richness (Ostoja, Schupp, & Sivy, 2009). In many cases, fire decreases the diversity of the entire insect community (Swengel, 2001).

The ability of rodents to modify plant community structure, and their sensitivity to fire, could result in rodent communities having important effects on invertebrate communities in postfire environments. Many rodent species include insects as part of their diet, and small mammal insectivory has been shown to have strong effects on grassland invertebrate communities (Churchfield, Hollier, & Brown, 1991). The effects of rodents on plant community structure via granivory and folivory (Sharp‐Bowman, McMillan, & St. Clair, 2017a, 2017b) are also likely to have indirect effects on the abundance and diversity of insect communities. A previous study at our Great Basin site determined that rodents can suppress cheatgrass invasion (St. Clair, O'Connor, Gill, & McMillan, 2016); rodent exclusion produced a plant community dominated by invasive grasses, and where rodents had access, the plant community was a much more diverse annual forb community. In both burned treatments (with and without rodents), plant diversity was reduced compared to unburned plots, but burned plots with rodent access had higher plant diversity than burned plots without rodents; these changes in plant habitat could have bottom‐up influences on invertebrate diversity. Banner‐tailed kangaroo (*Dipodomys spectabilis*) rats have been known to alter ant community composition (Schooley, Bestelmeyer, & Kelly, 2000) via changes in plant community structure through mound building (Moroka, Beck, & Pieper, 1982). The indirect effects of rodents on insect communities through the modification of the plant community are not well characterized. Our study was designed to increase the characterization of the indirect effects of rodents on insect communities.

There have been many studies in the deserts of North America documenting plant–insect interactions (Ostoja et al., 2009) and rodent–insect interactions (Brown & Davidson, 1977; Brown, Davidson, & Reichman, 1979), but there are far fewer studies that compared these relationships across different desert ecosystems. The deserts of western North America vary in climate and have unique biotic communities. The Great Basin and the Mojave Desert share a border but are very different from one another, one is semi‐arid while the other is hyperarid. Despite these differences, both are facing a similar threat of changing fire regimes caused by invasive annual grasses. Because of their inherent differences, the biological communities in each desert may respond differently to these changes. The purpose of our study was to characterize the influence of fire and rodent exclusion on invertebrate community abundance and diversity in the Great Basin and Mojave Deserts and whether they were related to changes in the plant community. This study addressed the following questions: (a) What are the impacts of fire and rodent exclusion on invertebrate communities and do the responses vary between the Mojave and Great Basin Deserts? (b) Do the abundances of different invertebrate groups respond similarly to fire and rodent exclusion? (c) How do fire and rodent exclusion affect the species richness and diversity of ant communities? (d) Are fire and rodent effects on invertebrates mediated by changes in the plant community?

## 2 METHODS

### 2.1 Study sites

#### 2.1.1 Great Basin

Our Great Basin site is located in Rush Valley in southeast Toole Co., Utah (40°05′27″N 112°18′18″W). Elevation is 1,650 m and mean annual temperature is 8.6°C, with an average mean January temperature of 3.2°C and an average mean July temperature of 22.3°C (Vernon GHCN:COOP, Utah Climate Center). The study site is dominated by Wyoming big sagebrush (*Artemesia tridentata wyomingensis*), and at the beginning of the experiment, only one other native plant was common, the perennial bunch grass bottlebrush squirreltail (*Elymus elymoides*). The most common invasive plant at this site was the winter annual cheatgrass (*Bromus tectorum*). There was little evidence of grazing and no evidence of fire in the last several decades prior to the start of the experiment in 2011. During the 2016 sampling period, April received the highest amount of precipitation with 4.83 cm, with May, June, July, and September receiving 1.44, 1.09, 1.68, and 2.02 cm, respectively (Vernon GHCN:COOP, Utah Climate Center).

#### 2.1.2 Mojave Desert

Our Mojave Desert site is located at the Lytle Ranch Preserve, which is a 680‐acre nature preserve owned and managed by Brigham Young University. Lytle Ranch is located in the northern Mojave Desert, in western Washington Co., Utah (37°08′54″N 114°00′50″W). Elevation is 915 m, mean annual temperature is 16.3°C, average mean January temperature is 6.2°C, and average mean July temperature is 28.1°C (Lytle Ranch GHCN, Utah Climate Center). Dominant plants in the study site are Joshua trees (*Yucca brevifolia*), creosote bush (*Larrea tridentata*), and blackbrush (*Coleogyne ramosissima*). The most common invasive plants were red brome (*Bromus rubens*) and Arabian schismus (*Schismus arabicus*), both annual grasses. There has been no grazing in the last 30 years and no evidence of fire in several decades. During the 2016 sampling period, April, July, and August were the only months to have precipitation, with those months receiving 4.96, 0.56, and 4.09 cm, respectively (Lytle Ranch GHCN:COOP, Utah Climate Center).

### 2.2 Experimental design

The experimental design at both sites was the same and consisted of 60 × 60 m experimental blocks replicated five times. Each block was split into four equal (30 × 30 m) subplots, which were assigned to one of four factorial treatment combinations: burned or unburned, and rodent access or rodent exclusion (St. Clair et al., 2016). Each site was protected from cattle by a barb‐wire fence, with enough room at the bottom to allow free movement of native wildlife. Rodent fences were established using 1 m tall welded‐wire fencing which was buried 30 cm below the soil surface so that it extended 70 cm above the surface. The two plots in each block that were randomly assigned the rodent exclusion treatment had 20 cm of smooth metal flashing attached to the top of the fence to prevent rodents from climbing over the top. The two remaining plots had 12 × 10 cm openings cut in the bottom of the fence every 4 m to allow rodents movement in and out of the plots. Rodent trapping sessions, conducted in April and July of 2016 at both the Mojave and Great Basin sites, revealed that rodent abundance was typical at the Mojave site and 50% higher than normal at the Great Basin site (Sharp‐Bowman et al.,2017a), and rodent fences reduced rodent abundance 2–3 fold.

For each of the five experimental blocks, one rodent exclusion and one rodent control plot were randomly selected and independently burned, completing the full factorial design. The burn treatments occurred in June 2011 at the Mojave site and in September 2011 at the Great Basin site. The fires were started with drip torches and resulted in high burn severity with a majority of the native plant cover removed (>90%). To facilitate the spread of fire between shrubs at the Great Basin site, we placed 300 g/m^−2^ of wheat straw in the shrub interspaces in our burn plots (St. Clair et al., 2016). Fire spread naturally without straw at the Mojave site.

### 2.3 Invertebrate trapping

There were 4 pitfall traps placed in each experimental subplot (Andersen, 1991), 10 m diagonally from each corner toward the center of the plot; each trap was 7.62 cm diameter. For each trapping session, traps were filled with approximately 90 ml of propylene glycol and left open for approximately 72 hr. At the end of each trapping session, the contents of the traps were collected and placed in 70% isopropyl alcohol for later sorting and identification. Trapping sessions were performed at each location once a month from April through September 2016, five years after the treatments were imposed. Invertebrates were identified to family, where possible, and ants were identified to species. To better understand effects of fire on the invertebrate community, we categorized them into four functional groups that varied in their foraging extent (ground dwellers, flying foragers, ground foragers, and ants). Flying foragers can easily select for unburned terrain, ground foragers less so, and ground dwellers are incapable. Ants comprised their own group due to high relative abundances and identification to species, which was not replicated in the other invertebrate samples. Foraging behavior was determined from natural history information in Borror and Delong's Introduction to the Study of Insects (Triplehorn & Johnson, 2005).We used the most abundant taxa from each group for our analysis. We selected taxa that were represented at both sites and had ≥four individuals. We excluded rare invertebrate families because it was impossible for us to determine whether they were simply rare in our system or rare because of our trapping method. The ground‐dwelling group across both sites comprised invertebrates from the taxa Acari, Entomobryidae, Sminthuridae, and Meinertellidae. Our flying‐forager group across both sites comprised from the taxa Sarcophagidae, Sphecidae, Anthomyiidae, Geocoridae, Phoridae, Cicadellidae, Sciaridae, Bethylidae, Hymenoptera, and Cecidomyiidae. The ground‐foraging group across both sites comprised from the taxa Tenebrionidae, Carabidae, Histeridae, Acrididae, Araneae, Scarabaeidae, Solifugae, Elateridae, and Rhaphidophoridae (Tables 1 and 2).

**Table 1:** Total numbers of individuals of each taxa for the Mojave site, with the taxa separated by functional group

Functional group/taxon	BRA	BRX	Total burned	URA	URX	Total unburned
Ground‐dweller						
Acari	482	586	1,068	703	870	1,573
Sminthuridae	22	13	35	9	62	71
Entomobryidae	146	281	427	269	180	449
Meinertellidae	11	5	16	15	19	34
Flying‐forager						
Cicadellidae	227	325	552	189	192	381
Anthomyiidae	17	8	25	7	18	25
Hymenoptera	15	31	46	29	31	60
Sciaridae	36	63	99	39	33	72
Bethylidae	30	25	55	26	12	38
Cecidomyiidae	41	43	84	78	134	212
Phoridae	53	62	115	79	59	138
Sphecidae	13	14	27	19	14	33
Sarcophagidae	10	4	14	12	23	35
Geocoridae	56	50	106	30	47	77
Ground‐forager						
Carabidae	9	22	31	17	7	24
Tenebrionidae	29	12	41	23	39	62
Acrididae	8	4	12	2	2	4
Rhaphidophoridae	13	19	32	31	16	47
Histeridae	23	18	41	42	9	51
Scarabaeidae	28	24	52	9	18	27
Solifugae	9	3	12	5	10	15
Elateridae	9	15	24	25	32	57

Note

Numbers are separated by treatment combination: Unburned‐rodent access, URA; unburned‐rodent exclusion, URX; burned‐rodent access, BRA; burned‐rodent exclusion, BRX. Numbers represent total amounts collected for 2016 season.

**Table 2:** Total numbers of individuals of each taxa for the Great Basin site, with the taxa separated by functional group

Functional group/taxon	BRA	BRX	Total burned	URA	URX	Total unburned
Ground‐dweller						
Acari	714	484	1,198	892	485	1,377
Sminthuridae	168	71	239	65	81	146
Entomobryidae	555	2,045	2,600	277	191	468
Meinertellidae	1	0	1	1	2	3
Flying‐forager						
Cicadellidae	75	84	159	109	127	236
Anthomyiidae	57	62	119	81	65	146
Hymenoptera	33	11	44	20	20	40
Sciaridae	18	14	32	9	81	90
Bethylidae	15	10	25	14	10	24
Cecidomyiidae	14	29	43	88	103	191
Phoridae	32	26	58	60	42	102
Sphecidae	25	26	51	12	13	25
Sarcophagidae	18	6	24	30	15	45
Geocoridae	42	18	60	5	2	7
Ground‐forager						
Carabidae	13	4	17	8	3	11
Tenebrionidae	13	7	20	13	2	15
Acrididae	11	24	35	23	32	55
Rhaphidophoridae	7	3	10	10	5	15
Histeridae	3	1	4	1	1	2
Scarabaeidae	2	1	3	1	1	2
Solifugae	4	8	12	4	8	12
Elateridae	0	0	0	3	0	3

Note

Numbers are separated by treatment combination: Unburned‐rodent access, URA; unburned‐rodent exclusion, URX; burned‐rodent access, BRA; burned‐rodent exclusion, BRX. Numbers represent total amounts collected for 2016 season.

Fluctuations in the abundances of dominant taxa have been shown to drive ecosystem services (Winfree, Fox, Williams, Reilly, & Cariveau, 2015). For this reason, we focused our analysis on the most abundant taxa that were represented at both sites. Because of the large spatial and temporal scale that we sampled and the specialized nature of identifying down to species, most invertebrates were identified to family and we only present abundance data. Ants all belong to the same family (Formicidae), and are established bioindicators of ecosystem function (Majer, 1983). Pitfall trapping is a well‐established method for capturing ants (Andersen, 1991). Ants are also relatively easy to identify and are common in most terrestrial ecosystems throughout the world. Therefore, we were able to identify ants to species and determine changes in richness and diversity of ant species in response to treatment conditions. Our results can also thus be readily compared to a wider range of studies.

### 2.4 Vegetation surveys

Vegetation cover and density were measured at both sites. Vegetation surveys were conducted in April 2016 at the Mojave site and in June 2016 at the Great Basin site. Cover was measured using the step point intercept method (Bonham, 1989). Four 30‐m transects were randomly placed parallel to each other in each plot. Starting at the two‐meter mark, a pin was dropped every 50 cm along each transect with a total of 46 points for each transect. Canopy, as well as first (next layer under canopy), second, and third foliar layers were recorded by species, and basal cover was also recorded where present. Cover measurements for each species for each plot were calculated by taking the total number of hits of each species across the four transects and dividing them by 184, and the resulting number was recorded as a percentage. In order to compare responses between sites, plant cover was separated into three groups for analysis: invasive herbaceous plants, native herbaceous plants, and shrubs.

### 2.5 Statistical analysis

We used repeated‐measures analysis of variance (ANOVA) models to test the main and interactive effects of fire and rodent exclusion across time (monthly samples) on the abundances of individuals for each of our three functional groups (ground dwellers, flying foragers, and ground foragers), as well as ant abundance, richness, and Shannon's diversity index. We used structural equation modeling (*SEM*) to estimate the indirect effects of fire and rodent exclusion, mediated through the plant community, on ant abundance, richness, and Shannon's diversity index, as well as on the abundances of our three invertebrate functional groups (Lefcheck, 2016). We ran structural equation modeling (*SEM*) using the R package “piecewiseSEM” (version 3.2.2 R Core Team, Vienna, Austria). We fit linear mixed effects models using the “nlme” package in R, and block was included as a random factor for each model (Pinheiro, Bates, DebRoy, Sarkar, & Team, 2016). We computed the conditional *R* ^2^ for each model using the method of Nakagawa and Schielzeth (2013). We log‐transformed ant abundance, ground‐dweller abundance, and flying‐forager abundance at both sites to meet model assumptions of normality and homogeneity of variance. We used a square‐root transformation of ground‐forager abundance at both sites to meet model assumptions. Repeated‐measures ANOVA models were calculated using the program JMP (SAS Institute Inc.). In running the statistical analyses, there were no significant main effects of rodent exclusion or rodent exclusion by fire interactions.

## 3 RESULTS

We identified 101 families or orders from the Great Basin site. We also identified 10 ant species representing nine genera in the Great Basin site. We identified 108 families or orders from the Mojave Desert site. We also identified twelve ant species representing nine genera in the Mojave Desert (Tables 3 and 4).

**Table 3:** Numbers of individual ant foragers of each species collected from the Mojave site, with the species separated by subfamily

Subfamily/species	BRA	BRX	Total Burned	URA	URX	Total Unburned
Dolichoderinae						
*Forelius pruinosus*	463	395	858	692	320	1,012
*Dorymyrmex pyramicus*	30	5	35	47	41	88
Formicinae						
*Myrmecocystus mexicanus*	80	25	105	205	306	511
*Myrmecocystus semirufus*	4	2	6	2	0	2
Myrmicinae						
*Monomorium ergatogyna*	18	0	18	1	1	2
*Tetramorium hispidum*	1	4	5	14	33	47
*Pheidole desertorum*	32	14	46	611	246	857
*Pheidole gilvescens*	209	280	489	286	187	473
*Pogonomyrmex rugosus*	1,787	2,414	4,201	795	681	1,476
*Solenopsis molesta*	0	3	3	0	0	0
*Solenopsis xyloni*	2,063	938	3,001	649	582	1,231
*Crematogaster depilis*	0	0	0	18	30	48

Note

Numbers are separated by treatment combination: Unburned‐rodent access, URA; unburned‐rodent exclusion, URX; burned‐rodent access, BRA; burned‐rodent exclusion, BRX. Numbers represent total amounts collected for 2016 season.

**Table 4:** Numbers of individual ant foragers of each species collected from the Great Basin site, with the species separated by subfamily

Subfamily/species	BRA	BRX	Total burned	URA	URX	Total unburned
Dolichoderinae						
*Forelius pruinosus*	81	73	154	200	125	325
Formicinae						
*Camponotus vicinus*	7	3	10	41	39	80
*Myrmecocystus hammettensis*	13	2	15	8	4	12
*Myrmecocystus testaceus*	2	2	4	4	57	61
Myrmicinae						
*Monomorium ergatogyna*	25	207	232	15	16	31
*Myrmica lobifrons*	0	10	10	7	6	13
*Pheidole jtl‐222*	4	19	23	4	7	11
*Pogonomyrmex occidentalis*	389	229	618	239	191	430
*Solenopsis molesta*	11	17	28	4	4	8
*Temnothorax nevadensis*	6	9	15	13	16	29

Note

Numbers are separated by treatment combination: Unburned‐rodent access, URA; unburned‐rodent exclusion, URX; burned‐rodent access, BRA; burned‐rodent exclusion, BRX. Numbers represent total amounts collected for 2016 season.

### 3.1 Fire effects

**Figure 1:**
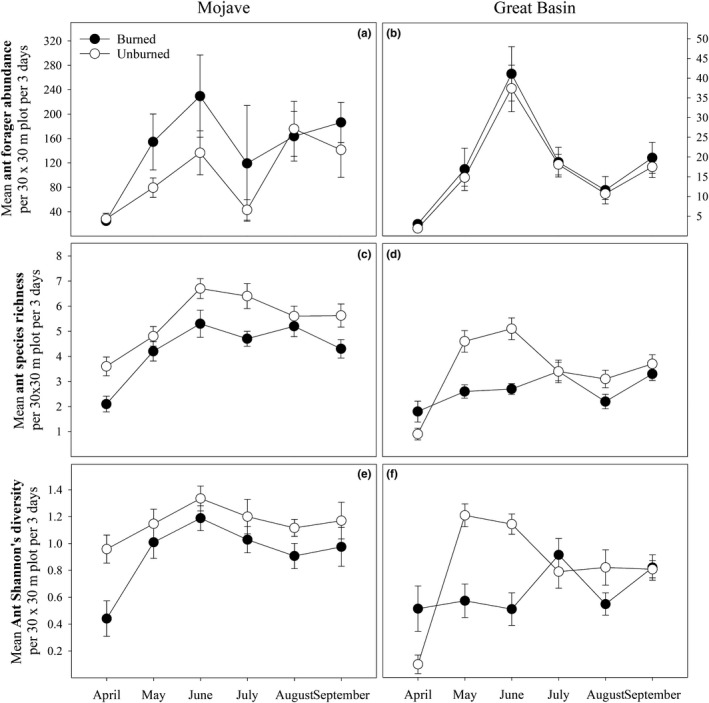
Ant forager abundance (a and b), species richness (c and d), and Shannon's diversity (e and f) responses to burn treatment separated by month and site. Error bars represent standard error for each value

**Figure 2:**
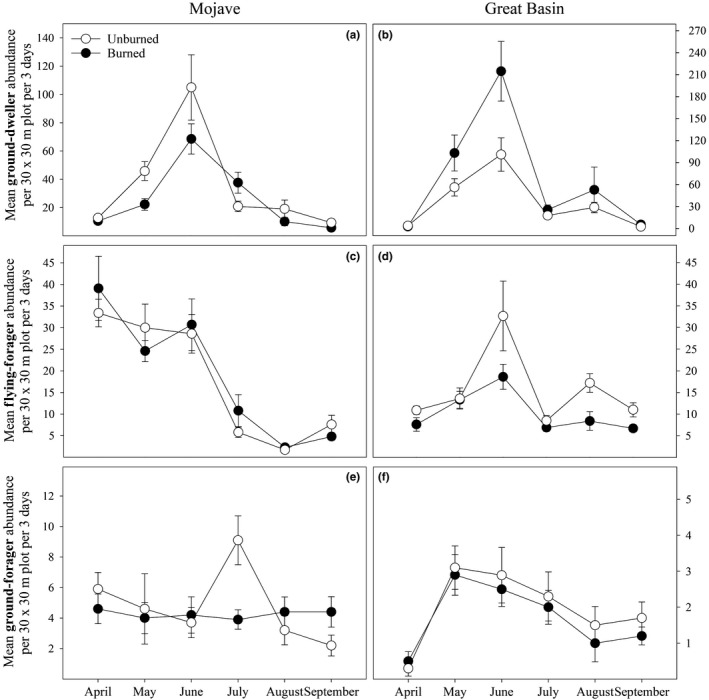
Ground‐dweller abundance (a and b), flying‐forager abundance (c and d), and ground‐forager abundance (e and f) responses to burn treatments separated by month and site. Error bars represent standard error for each value

**Figure 3:**
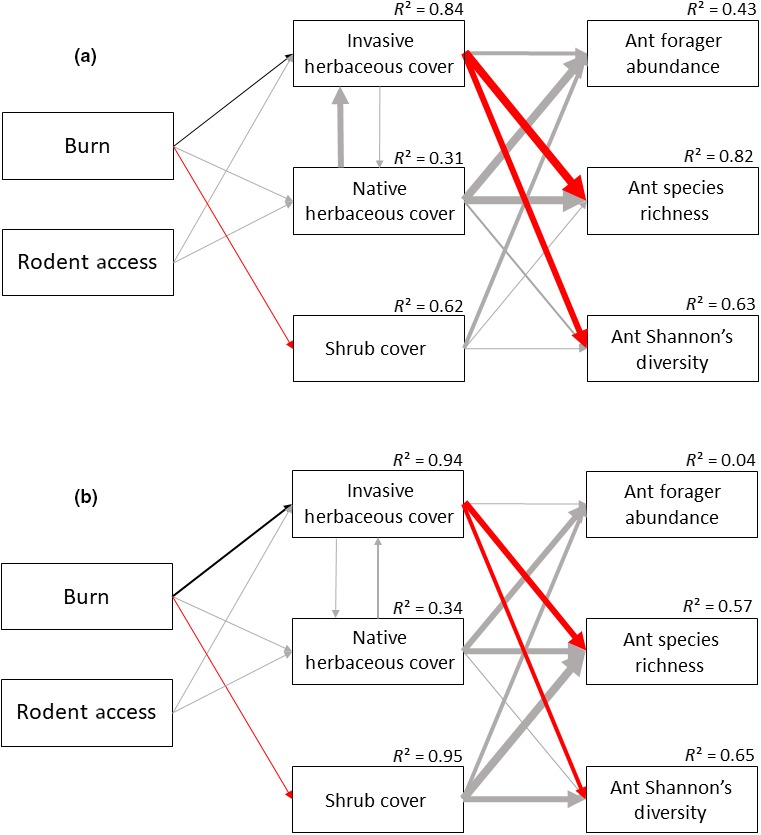
Structural equation models, showing relationships between burn treatments, rodent treatments, plant cover types and the forager abundance, species richness, and Shannon's diversity of the ant communities in the Mojave (a) and the Great Basin Deserts (b). *R* ^2^ values are shown for each model. Gray lines represent nonsignificant interactions (*p* > 0.05), black lines represent positive significant interactions (*p* ≤ 0.05), and red lines represent negative significant interactions (*p* ≤ 0.05). Line width indicates strength of interaction; thicker lines mean stronger interaction

**Figure 4:**
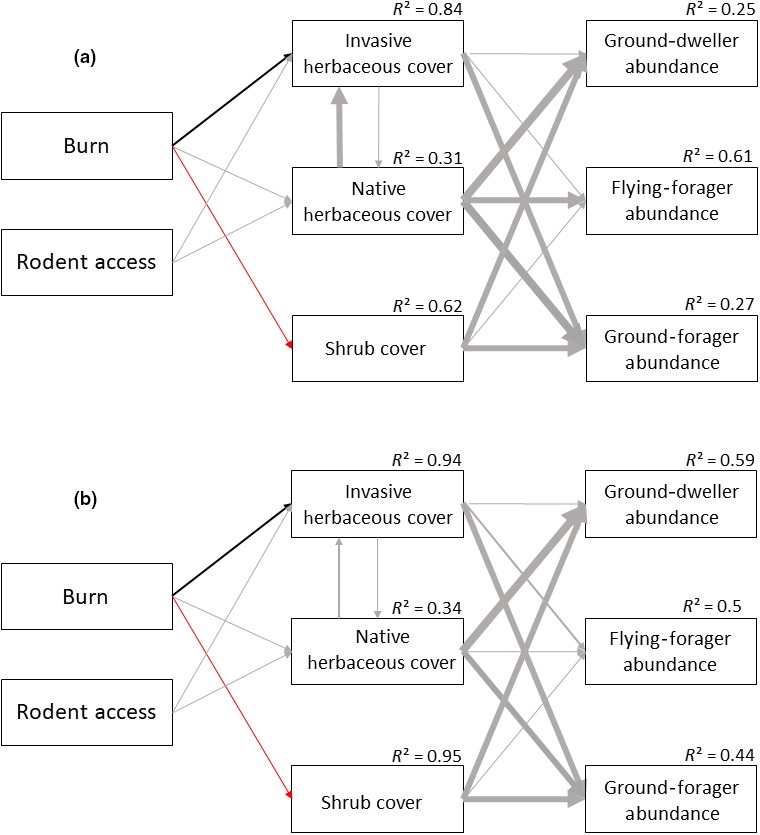
Structural equation models, showing relationships between burn treatments, rodent treatments, plant cover types and the ground‐dweller abundance, flying‐forager abundance, and ground‐forager abundance in the Mojave (a) and the Great Basin Deserts (b). *R* ^2^ values are shown for each model. Gray lines represent nonsignificant interactions (*p* > 0.05), black lines represent positive significant interactions (*p* ≤ 0.05), and red lines represent negative significant interactions (*p* ≤ 0.05). Line width indicates strength of interaction; thicker lines mean stronger interaction

Fire affected ant species richness and diversity at both sites (Tables 5 and 6), with higher species richness and diversity in unburned plots than in burned plots (Figure 1). In the Great Basin, the effect of fire on ant diversity was only significant in May and June (Table 6; Figure 1). Fire did not significantly affect ant abundance at either site (Tables 5 and 6). Fire also had little effect on the abundances of ground dwellers or ground foragers at either site (Tables 5 and 6). Fire played a significant role in determining flying‐forager abundance at the Great Basin site (Table 6), with higher flying‐forager abundance in unburned areas than in burned areas (Figure 2), but fire had little effect on flying‐forager abundance at the Mojave site (Table 5). Structural equation models suggest that the effects of fire on ant species richness and Shannon's diversity at both sites are mediated through changes in the plant communities (Tables 7 and 8; Figure 3). Specifically, fire had a positive influence on invasive plant cover, which then negatively influenced ant species richness and diversity (Tables 7 and 8; Figure 3). Structural equation models did not show any effects of plant cover on ground‐dweller, flying‐forager, or ground‐forager abundance at either site (Tables 7 and 8; Figure 4).

**Table 5:** *F* values from repeated measures ANOVA models for Mojave Desert invertebrate functional group and ant community responses to treatments

Treatments	Ant forager abundance	Ant species richness	Ant Shannon's diversity	Ground‐dweller abundance	Flying‐forager abundance	Ground‐forager abundance
Mojave Desert						
Fire	0.6	**11** **	**7.1** *	3.1	0.1	0.3
Rodents	0.1	1.0	0.1	0.1	0.1	0.4
Month	**19** ***	**24** ***	**6.5** **	**49** ***	**63** ***	3.2
Fire ×Rodents	0.0	0.2	0.4	0.6	0.6	0.1
Fire ×Month	1.7	1.8	0.5	2.7	0.2	1.2
Rodents × Month	0.6	1.1	0.8	3.1	0.4	0.3
Fire ×Rodents ×Month	0.4	0.2	0.2	1.6	0.5	1.3

Note

Bold = significant at *p* < 0.1

* *p* < 0.05.

** *p* < 0.01.

*** *p* < 0.001.

**Table 6:** *F* values from repeated measures ANOVA models for Great Basin invertebrate and ant community responses to treatments averaged for the whole year

Treatments	Ant foraging abundance	Ant species richness	Ant Shannon's diversity	Ground‐dweller abundance	Flying‐forager abundance	Ground‐forager abundance
Great Basin Desert						
Fire	0.1	**7.7** *	3.9	1.3	**15** **	0.4
Rodents	0.3	0.1	0.0	0.6	0.0	0.2
Month	**41** ***	**12** **	**7.5** *	**34** ***	**9.5** **	**24** ***
Fire × Rodents	0.7	0.0	0.1	**4.9** *	0.8	0.0
Fire × Month	1.0	**7.5** *	**8.8** *	1.4	1.4	0.6
Rodents × Month	**4.1** *	1.9	1.1	0.2	0.2	2.1
Fire × Rodents × Month	2.4	1.6	1.2	1.5	0.6	0.6

Note

Bold = significant at *p* < 0.1

* *p* < 0.05.

** *p* < 0.01.

*** *p* < 0.001.

**Table 7:** Path estimates, standard error, and *p*‐value for Mojave Desert structural equation models

Response	Predictor	Estimate	Std. error	*p* Value
Invasive herbaceous cover	Burn	0.188	0.031	**0.000**
Invasive herbaceous cover	Rodent access	0.019	0.031	0.554
Invasive herbaceous cover	Native herbaceous cover	3.470	2.332	0.163
Native herbaceous cover	Burn	0.003	0.005	0.567
Native herbaceous cover	Rodent access	−0.006	0.003	0.082
Native herbaceous cover	Invasive herbaceous cover	0.016	0.016	0.361
Shrub cover	Burn	−0.159	0.031	**0.000**
Ant Shannon's diversity	Invasive herbaceous cover	−1.090	0.304	**0.004**
Ant Shannon's diversity	Native herbaceous cover	−0.516	4.583	0.912
Ant Shannon's diversity	Shrub cover	0.257	0.407	0.539
Ant species richness	Invasive herbaceous cover	−5.098	1.309	**0.002**
Ant species richness	Native herbaceous cover	−19.261	16.142	0.256
Ant species richness	Shrub cover	−0.296	1.519	0.849
Ant forager abundance	Invasive herbaceous cover	0.881	1.248	0.494
Ant forager abundance	Native herbaceous cover	−10.372	16.103	0.532
Ant forager abundance	Shrub cover	−0.958	1.495	0.534
Ground‐dweller abundance	Invasive herbaceous cover	−0.066	1.095	0.953
Ground‐dweller abundance	Native herbaceous cover	13.395	14.903	0.386
Ground‐dweller abundance	Shrub cover	1.257	1.363	0.375
Flying‐forager abundance	Invasive herbaceous cover	0.040	0.586	0.946
Flying‐forager abundance	Native herbaceous cover	1.131	6.975	0.874
Flying‐forager abundance	Shrub cover	0.006	0.663	0.994
Ground‐forager abundance	Invasive herbaceous cover	1.050	0.916	0.274
Ground‐forager abundance	Native herbaceous cover	−14.541	11.697	0.238
Ground‐forager abundance	Shrub cover	1.088	1.089	0.338

Note

*p* Values <0.05 are bolded for emphasis.

**Table 8:** Path estimates, standard error, and *p*‐value for Great Basin Desert structural equation models

Response	Predictor	Estimate	Std. error	*p* Value
Invasive herbaceous cover	Burn	0.568	0.038	**0.000**
Invasive herbaceous cover	Rodent access	−0.052	0.037	0.190
Invasive herbaceous cover	Native herbaceous cover	−0.548	0.638	0.407
Native herbaceous cover	Burn	−0.009	0.052	0.864
Native herbaceous cover	Rodent access	−0.027	0.014	0.085
Native herbaceous cover	Invasive herbaceous cover	−0.034	0.086	0.703
Shrub cover	Burn	−0.196	0.010	**0.000**
Ant Shannon's diversity	Invasive herbaceous cover	−0.818	0.324	**0.026**
Ant Shannon's diversity	Native herbaceous cover	0.147	0.946	0.879
Ant Shannon's diversity	Shrub cover	−1.620	0.939	0.110
Ant species richness	Invasive herbaceous cover	−2.879	1.267	**0.042**
Ant species richness	Native herbaceous cover	4.320	3.699	0.266
Ant species richness	Shrub cover	−5.331	3.693	0.175
Ant forager abundance	Invasive herbaceous cover	0.437	0.874	0.626
Ant forager abundance	Native herbaceous cover	1.465	2.548	0.576
Ant forager abundance	Shrub cover	0.917	2.566	0.727
Ground‐dweller abundance	Invasive herbaceous cover	−0.349	1.118	0.760
Ground‐dweller abundance	Native herbaceous cover	−6.380	3.269	0.075
Ground‐dweller abundance	Shrub cover	−3.712	3.252	0.276
Flying‐forager abundance	Invasive herbaceous cover	−0.571	0.653	0.399
Flying‐forager abundance	Native herbaceous cover	0.462	1.908	0.813
Flying‐forager abundance	Shrub cover	0.405	1.907	0.835
Ground‐forager abundance	Invasive herbaceous cover	−1.396	0.761	0.091
Ground‐forager abundance	Native herbaceous cover	1.152	2.222	0.614
Ground‐forager abundance	Shrub cover	−3.250	2.215	0.168

Note

*p* Values <0.05 are bolded for emphasis.

### 3.2 Rodent effects

**Figure 5:**
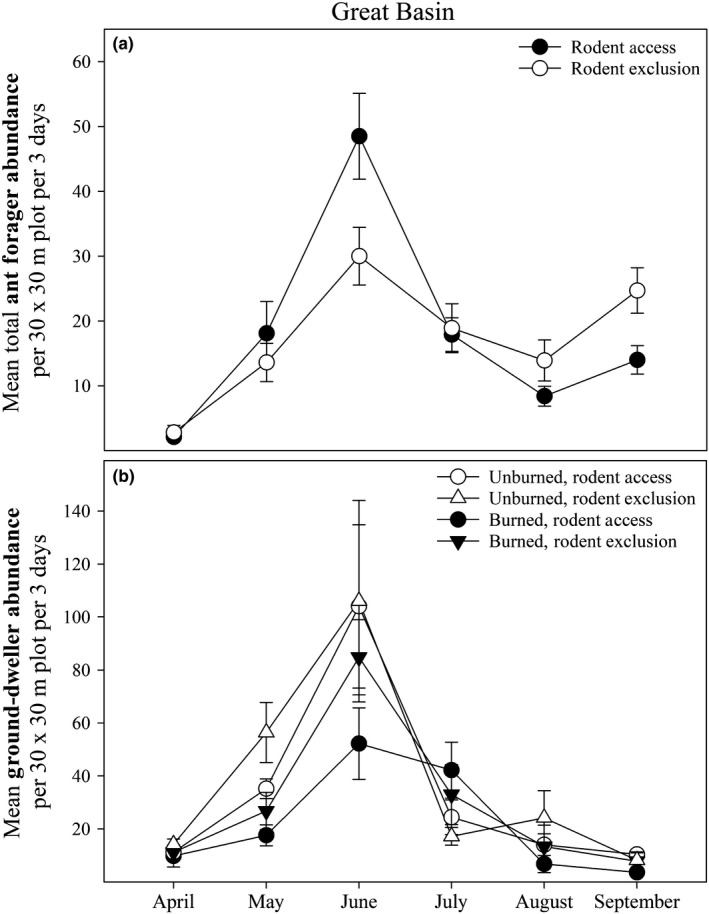
Responses of ant forager abundance to rodent treatment (a) and ground‐dweller abundance to fire and rodent treatments (b) separated by month in the Great Basin. Error bars represent standard error for each value

Rodent treatments had little to no effect on ant abundance, richness, or diversity at either location when averaged across months (Tables 4 and 5). At our Great Basin site, rodents had a significant effect on ant abundance depending on the month, with abundance being higher in rodent access plots than in rodent exclusion plots in May and June but being lower in rodent exclusion plots in August and September (Table 6; Figure 5). Rodent treatments had little to no effect on flying‐forager abundance or ground‐forager abundance at either site (Tables 5 and 6).

### 3.3 Fire and rodent interactions

Fire and rodent interaction terms in our models were generally not significant (Tables 5 and 6). The only exception to this was the abundance of ground‐dweller invertebrates at our Great Basin site (Table 6), where abundance was higher in rodent exclusion plots, particularly in unburned conditions (Figure 5).

### 3.4 Time effects

Ant abundance, species richness, and Shannon's diversity at both locations changed significantly across months (Tables 5 and 6), with abundance and species richness peaking in June at both locations (Figure 1). Ant diversity was highest in June in the Mojave and highest in May in the Great Basin (Figure 1). At the Mojave site, ground‐dweller abundance and flying‐forager abundance changed significantly across time (Table 5), with ground‐dweller abundance peaking in June and flying‐forager abundance being highest in April (Figure 2). At the Great Basin site, ground‐dweller abundance, flying‐forager abundance, and ground‐forager abundance were all significantly affected by month (Table 6), with ground‐dweller and flying‐forager abundances peaking in June, and ground‐forager abundance peaking in May (Figure 2).

## 4 DISCUSSION

### 4.1 Invertebrate responses to fire

Fire can have both positive and negative effects on invertebrate abundance and diversity (Swengel, 2001), with the effects varying depending on the taxa measured and other environmental conditions (Warren, Scifres, & Teel, 1987). In our study, fire had very little effect on ant abundance; however, fire reduced ant species diversity at both sites (Tables 5 and 6; Figures 1 and 2). Ostoja et al. (2009) reported lower ant diversity in cheatgrass‐dominated plots compared with sagebrush intact plots in the Great Basin which is a typical vegetation conversion after fire as seen in our plots. These results are consistent with our previous research at the Great Basin site, where ant species diversity was reduced in burned areas but ant abundance was unaffected (Day, Bishop, & St. Clair, 2018). In that study, abundance of most species was reduced in burned plots, but the abundances of some dominant ant species increased, which kept overall ant abundance in burned areas similar to those in unburned areas. This same pattern occurred at our Mojave site, where reduction in abundance for some ant species was balanced by the increase in abundance of others (Table 3). Among the dominant ant species that responded positively to fire were harvester ants in the genus *Pogonomyrmex* (Tables 3 and 4). Holbrook et al. (2016) reported increased *P. occidentalis* nest density in burned areas over unburned areas in the Great Basin. Our surveys show that *P. occidentalis* forager abundance increased in burned plots at our Great Basin site (Table 4) while *P. rugosus* forager abundance at our Mojave site nearly tripled in burned plots compared to unburned plots (Table 3). This increase in *Pogonomyrmex* abundance may be the result of shrub removal, allowing for increased colony densities (Day et al., 2018; Sneva, 1979), or it may also be caused by increased seed resources from increases in annual plant cover.

The abundances of most of the invertebrate groups in our study were unaffected by fire (Tables 5 and 6; Figures 1 and 2). We did observe that the flying‐forager group saw reductions in abundance in burned plots compared to unburned plots in the Great Basin (Table 6; Figure 2), which may be related to their avoidance of burned habitat. Gall midge (Diptera: Cecidomyiidae) larvae feed within plant tissues and some may have specific associations with native plants that are lost during fires. Welch (2005) reported 32 species of midges that induce galls on *A. tridentata*, so shrub removal of sagebrush may reduce host plant availability. Harper, Dietrich, Larimore, and Tessene (2000) observed reductions in some leafhoppers (Hemiptera: Cicadellidae) in burned areas. Pitfall trapping is not a reliable method for sampling flying insects, so we were surprised to have collected so many. Our findings in this study suggest further research is needed on the effects of fire on flying insects in the Great Basin.

### 4.2 Indirect effects of fire mediated through changes in plant communities

Vegetation structure and plant community composition are important determiners in invertebrate community composition (Bromham, Cardillo, Bennett, & Elgar, 1999; Denno et al., 2002; Herrera & Dudley, 2003; Pearson, 2009). Lower ant diversity in burned plots may be a response to reduced resource availability or unfavorable abiotic conditions as a result of an altered plant community. Ant species richness and diversity were negatively influenced by invasive plant cover at both sites (Tables 7 and 8; Figure 3). This is consistent with findings of Ostoja et al. (2009) who found that ant diversity decreased in *B. tectorum‐*dominated sites compared to sagebrush intact sites in the Great Basin. Invasive plants were also reported to reduce ant species richness in a grassland (Lenda, Witek, Skorka, Moron, & Woyciechowski, 2013). Ants are generally thermophilic, but have varying levels of temperature tolerance (Hölldobler & Wilson, 1990). In sagebrush systems, shrub removal increases soil surface temperature (Chambers & Linnerooth, 2001) and reduces soil moisture in surface soils (Inouye, 2006). This change in abiotic conditions may favor some ant species, such as *Pogonomyrmex* (Bucy & Breed, 2006), but may restrict foraging time for other ant species. The abundances of arboreal ant species were reduced in burned plots at both sites; *Crematogaster depilis* in the Mojave was not found at all in burned areas during our study (Table 3), and *Camponotus vicinus* in the Great Basin was eight times more abundant in unburned plots than in burned plots during our study (Table 4). The life histories of more arboreal ants such as *C. depilis* and *C. vicinus* are closely tied to woody plants (Hölldobler & Wilson, 1990), which are greatly reduced in burned plots. The carpenter ant, *C. vicinus,* was reported to stop foraging when temperatures reach 23°C (Bernstein, 1979), so more shaded unburned areas may allow longer foraging times in summer. Nocturnal nectivorous ants, which may rely more on perennial plants for nectar resources, were also reduced in burned areas compared to unburned areas, *Myrmecocystus mexicanus* in the Mojave (Table 3) and *M. testaceus* in the Great Basin (Table 4).

### 4.3 Invertebrate responses to rodent exclusion

Rodents can have strong top‐down effects on Great Basin and Mojave plant communities (Sharp‐Bowman et al.,2017a, 2017b). Previous research in our Great Basin plots shows that rodent exclusion in burned areas dramatically increased the cover of cheatgrass leading to loss of plant biodiversity (St. Clair et al., 2016). We therefore expected to see top‐down effects of rodents on plant communities translate to shifts in invertebrate community composition and structure. However, we observed no significant main effects of rodent exclusion on ant community richness and diversity or invertebrate community abundance (Tables 5 and 6). Our results suggest that invasive plant cover strongly affects ant diversity (Tables 7 and 8; Figure 3), and while rodents may alter which types of invasive plants dominate in burned areas (St. Clair et al., 2016), they seem to have less effect on the percent cover of invasive plants in burned plots (Tables 7 and 8; Figure 3). For example, invasive plant cover in burned‐rodent access (BRA) and burned‐rodent exclusion (BRX) plots were nearly identical between sites (68% and 67% in BRA plots in the Mojave and Great Basin, respectively, and 72% in BRX plots at both sites). This suggests that the loss of native shrubs and their replacement by invasive annuals following fire has a larger impact on invertebrate communities than differences in the composition of invasive annual communities (annual grasses vs. annual forbs) created by rodents (St. Clair et al., 2016). Abiotic changes associated with shifts from native perennial shrublands to invasive annual plant communities, to which invertebrates are sensitive, are likely much greater than differences between invasive annual grass and annual forb communities.

### 4.4 Invertebrate responses over time

Seasonality played a significant role on invertebrate abundance and diversity in the Mojave (Table 5) and Great Basin (Table 6). In the Great Basin, the flying‐forager functional group exhibited a more sustained abundance throughout the sampling season in unburned areas, as opposed to a late season decline in burned areas. We also see similar patterns in the abundances of ground dwellers and ants over time at both sites. These effects are likely related to altered abiotic conditions favoring certain life‐history strategies. It is possible that altered vegetation dynamics could lead to different foraging patterns across seasons. For example, at our Great Basin site, leafhoppers exhibited a shift from more consistent abundance throughout the year in unburned areas, to a more concentrated abundance at the beginning of the year in burned areas. Insect abundance and presence have previously been linked with plant architecture (Stinson & Brown, 1983). After a burn, annual grasses can quickly fill open space and make the system more flammable as they quickly dry out toward the end of spring (Knapp, 1996). Changes in the seasonality of the vegetation likely alter the site selection of flying foragers, such as leafhoppers, that utilize the area for forage or laying eggs (Stinson & Brown, 1983).

Ant–plant interactions vary over seasons due to abiotic factors affecting both plants and ants (Rico‐Gray, Diaz‐Castelazo, Ramirez‐Hernandez, Guimaraes, & Holland, 2012). Temperature and precipitation are known to drive flammable changes in ant species richness and ant–plant interactions (Kaspari, O'Donnell, & Kercher, 2000; Rico‐Gray et al., 1998). Ant communities vary over the course of a year with changing plant phenology and may alternate food resources depending on the season (Rico‐Gray, 1993). The peak in ant forager abundance in early summer at both sites (Figure 1) is likely influenced by seed production, as most annual plants have gone to seed by June (Gordon, Holmes, & Nacu, 2008). The peaks in ant species richness and Shannon's diversity in May and June in unburned plots in the Great Basin (Figure 1) may be related to flowering events, seed production (Pol, Casenave, & Pirk, 2011), nectar production (Dáttilo et al., 2015), and increasing summer temperatures (Crist & MacMahon, 1991). The Mojave site saw higher Shannon's diversity in unburned plots every month, but total abundance was significantly higher in burned plots in May, June, and July (Figure 1). The Mojave site has much higher shrub diversity than the Great Basin site, so burned plots may be seeing more losses in ant‐shrub mutualisms (like that of *C. depilis *and cacti (Chamberlain & Holland, 2008)), lowering diversity throughout the study.

### 4.5 Desert comparison

Ants in general responded similarly to treatments at both sites when averaged over the whole collecting season; however, the way those responses played out over time differed between sites. In the Mojave, ant richness and diversity were higher in unburned than in burned plots throughout the entire collecting season (Figure 1). In the Great Basin, however, differences in ant richness and diversity between burn treatments were more variable (Figure 1). The Mojave site has higher mean temperatures than our Great Basin site through most of the year, and plant diversity is much higher at our Mojave site than at our Great Basin site. Ant foraging rates are dependent on both temperature (Crist & MacMahon, 1991; MacKay & MacKay, 1989) and food availability (Gordon et al., 2008). The higher plant diversity and higher mean temperatures in the Mojave may allow for the sustained difference in ant richness and diversity through the season.

## 5 IMPLICATIONS

Exotic invasive plants are changing desert fire regimes (D'Antonio & Vitousek, 1992), and their downstream impacts on invertebrate communities can have important ecological consequences. Fire facilitates invasion (Brooks et al., 2004) and invasion in turn facilitates fire (Balch, Bradley, D'Antonio, & Gomez‐Dans, 2013), creating a positive feedback loop and threshold resulting in potential state changes. Our data suggest that invertebrate community abundance is generally stable in response to desert fires but that species and taxonomic groups can vary dramatically. Invasive grass fire cycles pose a serious threat to arid systems where we may see significant modification to ecosystem function (Hooper et al., 2005), and perhaps local extinctions of some species. Biodiversity is already lower in arid systems than in more mesic systems because of abiotic limitations, and many of the species are operating near tolerance limits. This makes functional redundancy less likely in arid systems, which increases the importance of each invertebrate species in the system (Whitford, 1996). Our results show reductions in ant diversity in response to fire, suggesting potential losses of key ecosystem services tied to invertebrate diversity due to increases in fire frequency and extent. Even within a genus, there are important physiological, behavioral, and life‐history differences between species that minimize competition and affect the role that the individual species play in the ecosystem (Whitford, 1976, 1978). The replacement of less common species with more common ones may not adequately replace the services provided by the less common species. Fires and exotic annual grass invasion are combining to change the world for invertebrates in desert ecosystems.

## CONFLICT OF INTEREST

None declared.

## AUTHOR CONTRIBUTIONS

JDD collected samples, identified ants, analyzed data, and wrote the majority of the manuscript. JHB and TJT identified invertebrates, assisted with data analysis, and assisted in writing and editing the manuscript. SBB conceived and financially supported the study, directed analysis, and provided input and revisions during the writing process. PA contributed to several discussions related to ants and reviewed multiple drafts of the manuscript. AC collected and analyzed insect samples and data.

## DATA ACCESSIBILITY

Data available through the Dryad data repository: https://doi.org/10.5061/dryad.m171n70.
